# *Cephalotoma patcharinae* n. sp.—The First Record of *Cephalotoma* Species with a 2-Segmented Club of Antennae in the Oriental Region (Bostrichidae, Lyctinae: Trogoxylini) †

**DOI:** 10.3390/insects16010091

**Published:** 2025-01-16

**Authors:** Jerzy Borowski, Adam Byk, Sławomir Mazur, Tomasz Mokrzycki, Artur Rutkiewicz, Henryk Tracz, Agnieszka Ostrowska, Tomasz Oszako

**Affiliations:** 1 Department of Forest Protection, Institute of Forest Sciences, Warsaw University of Life Sciences SGGW, ul. Nowoursynowska 159/34, 02-766 Warsaw, Poland; adam_byk@sggw.edu.pl (A.B.); slawomir_mazur@sggw.edu.pl (S.M.); tomasz_mokrzycki@sggw.edu.pl (T.M.); artur_rutkiewicz@sggw.edu.pl (A.R.); henryk_tracz@sggw.edu.pl (H.T.); 2 Department of Nanobiotechnology, Institute of Biology, Warsaw University of Life Sciences SGGW, ul. Ciszewskiego 8, 02-786 Warsaw, Poland; agnieszka_ostrowska@sggw.edu.pl; 3 Faculty of Civil and Environmental Engineering, Bialystok University of Technology, Wiejska 45 A, 15-351 Bialystok, Poland; t.oszako@pb.edu.pl

**Keywords:** powderpost beetles, new species, new synonym, key for the Oriental species, *Cephalotoma*, Bostrichidae, Thailand

## Abstract

The paper presents a description of a new species of the genus *Cephalotoma* Lesne, caught in northern Thailand. The new species, *C. patcharinae* Borowski n. sp., is the first species in the Oriental region with a two-segmented club of antennae. The effect of the discussion presented in the paper is synonymising the tribe Cephalotomini Liu and Schönitzer, 2011 n. syn. with Trogoxylini Lesne, 1921. Moreover, the paper contains a key to classify all the species known from the Oriental region and belonging to the genus *Cephalotoma*.

## Simple Summary

The paper contains data of a newly discovered species of the powderpost beetles family, which was caught in northern Thailand. The new species, described and presented in photos, has a two-segmented club of antennae, a novelty among all the known species related to it, which occur in the Oriental region. Additionally, the paper includes a diagnosis, which contains morphological comparisons of the new species with other Asian species. The included discussion presents the characteristics of the tribe the new species belongs to; for the listed 12 characteristics of the tribe, comments and remarks are presented. The presented analysis clearly shows that the recently described tribe was wrongly defined. The final effect of the discussion is synonymising the wrongly defined tribe. The final part of the paper includes a key for the identification of all known species occurring in the Oriental region and belonging to the same genus as the newly described species.

## 1. Introduction

The tribe Trogoxylini Lesne, 1921, contains 25 species of 5 genera, occurring on all continents [1,2,3]. The most numerous genus, *Trogoxylon* LeConte, 1862, includes over half of the species of the tribe, but the Oriental fauna is represented by only two species: *T. spinifrons* (Lesne, 1910) and *T. auriculatum* Lesne, 1932 [1,2,3,4,5]. Also, in the described region, three species were reported, often brought in with timber, which represented other zoogeographic regions such as the Neotropical: *T. praeustum* (Erichson, 1847), Australian: *T. punctipenne* (Fauvel, 1904) or Cosmopolitan: *T. aequale* (Wollaston, 1867) [1,5]. The second genus in terms of the number of described species is *Cephalotoma* Lesne, 1911, represented worldwide by six species. Four of them occur in the Oriental region, and two in the Ethiopian region [3,6,7,8,9].

All of the species of the genus *Cephalotoma* are morphologically very similar to each other. The basic morphological feature differentiating the African and Oriental species was the morphology of the club of antennae, which has two segments in the former ones and three in the Oriental species. For this reason, Lesne [7] classified the African species into a separate genus and named it *Lyctoderma* Lesne, 1911. In 2012, the genus *Lyctoderma* became synonymous [2] with the genus *Cephalotoma*.

The images of the species of the genus *Cephalotoma* do not bite into wood to breed, as do most species of the family, but only use holes and galleries freshly bitten by other bostrichid beetles, e.g., of the genera *Xylothrips* Lesne, 1901; *Sinoxylon* Duftschmid, 1825; *Bostrychopsis* Lesne, *Bostrychoplites* Lesne, 1899; *Heterobostrychus* Lesne, 1899; or *Apate* Fabricius, 1775. The information about biting their own galleries [5] is highly doubtful for a simple reason, the morphology of images does not enable it. In the galleries bitten by other bostrichids, often even a dozen specimens of *Cephalotoma* can be found. However, it still has not been explained whether the representatives of *Cephalotoma* destroy the eggs of other powderpost beetles or just use a convenient microhabitat on the principle of commensalism [10].

Below, we present a description of the new species of the genus *Cephalotoma*, which was discovered in the northern part of Thailand. It is also the first Oriental species of the genus *Cephalotoma* with a two-segmented club of antennae.

## 2. Materials and Methods

With cooperation between the Department of Entomology and Plant Pathology, Chiang Mai University and the Department of Forest Protection IFS SGGW, Warsaw, during a scientific expedition in 2013, a new species of the genus *Cephalotoma* was caught in the northern part of Thailand.

Types are deposited in the collections of the Department of Forest Protection, SGGW, Warsaw, Poland.

Scanning photographs (SEM) were taken at the Institute of Zoology, Polish Academy of Science PAN, Warsaw and at Warsaw University of Life Sciences. Macrophotographs were taken with a Canon D60 (Tokyo, Japan) and processed with the licenced Helicon programme.

## 3. Results

### 3.1. Description of the New Species

**Figure 1:**
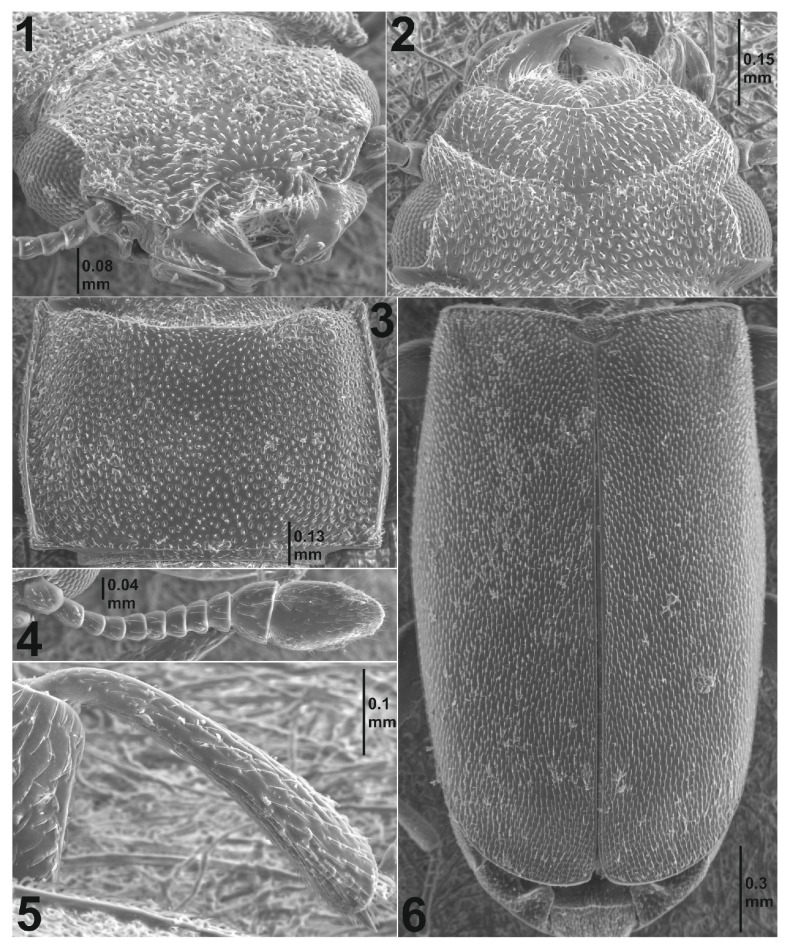
*Cephalotoma patcharinae* Borowski n. sp.: (**1**,**2**) head; (**3**) pronotum; (**4**) antenna; (**5**) front tibia; (**6**) elytra; (**1**,**5**) semilateral view; (**2**–**4**,**6**) dorsal view.

*Cephalotoma patcharinae* (Borowski) n. sp. (Figure 1(1–6)).

**Holotype**, ♂, NW Thailand, Khun Chang Khian, 98°53′56″ E, 18°50′17″ N, Highland Research Station, 4–6.III.2013, leg. J. Borowski; **Paratypes**, 3, ♀♀, 1♂, same data as the holotype.

Description. Length 2.5–3.8 mm. Body flattened, colour fuscous to dark fuscous, poorly shining. Labrum at the apical margin, in its central part, narrowly and deeply notched. Front edge of clypeus arched. Head short-haired; short setae, directed at labrum and clypeus (Figure 1(1,2)). Antennae of 11 segments, ending with 2-segmented club; the last segment of club of antennae is elongated, twice as long as the penultimate segment (Figure 1(4)). Pronotum transverse. Anterior margin of pronotum slightly arched; front angles of pronotum pointed and distinctly prolonged to the front. Pronotum sides with a distinct margin, slightly curved (Figure 1(3)). Pronotum base is straight, with a slight margin. Disc of pronotum with a distinct wide median depression. Scutellum trapezoid-shaped. Basal margin of elytra distinct at full length from humeri to scutellum. Elytra elongated, the widest in their central part, broadly curved at apices (Figure 1(6)). Surface of elytra slightly elevated in the posterior half, particularly near suture. Elytral punctures irregularly distributed; punctures small and densely spread. Elytral setation conspicuous. Setae dense, short, slightly upright, and directed at apices of elytra. Legs yellow-fuscous, sometimes femora are darker in central part. Front tibiae with single short apical spurs on the inside (Figure 1(5)). The last segment of tarsi ended with long claws. Ventral part of the body dark fuscous; lateral edges of abdomen sternites lighter. No clear sexual dimorphism.

### 3.2. Diagnosis

**Figure 2:**
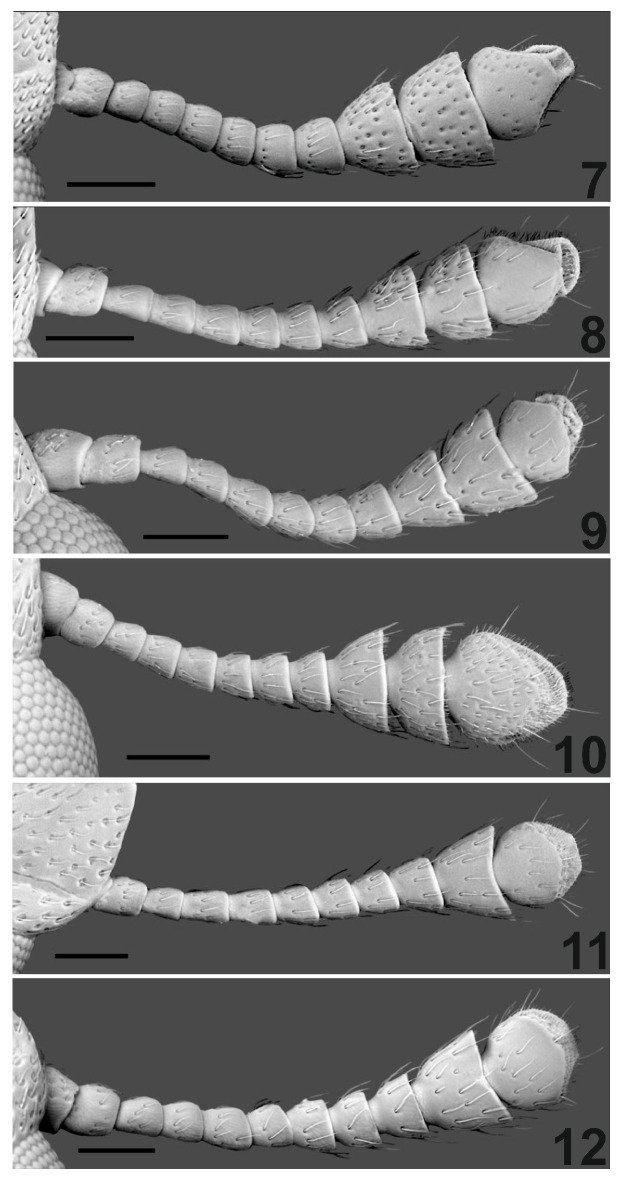
Antennae of *Cephalotoma* species. (**7**) *C. perdepressa* Lesne; (**8**) *C. singularis* Lesne; (**9**) *C. coomani* (Lesne); (**10**) *C. ambiguum* (Lesne); (**11**) *C. africanum* (Grouv.); (**12**) *C. testaceum* (Lesne). Scale = 0.1 mm.

**Figure 3:**
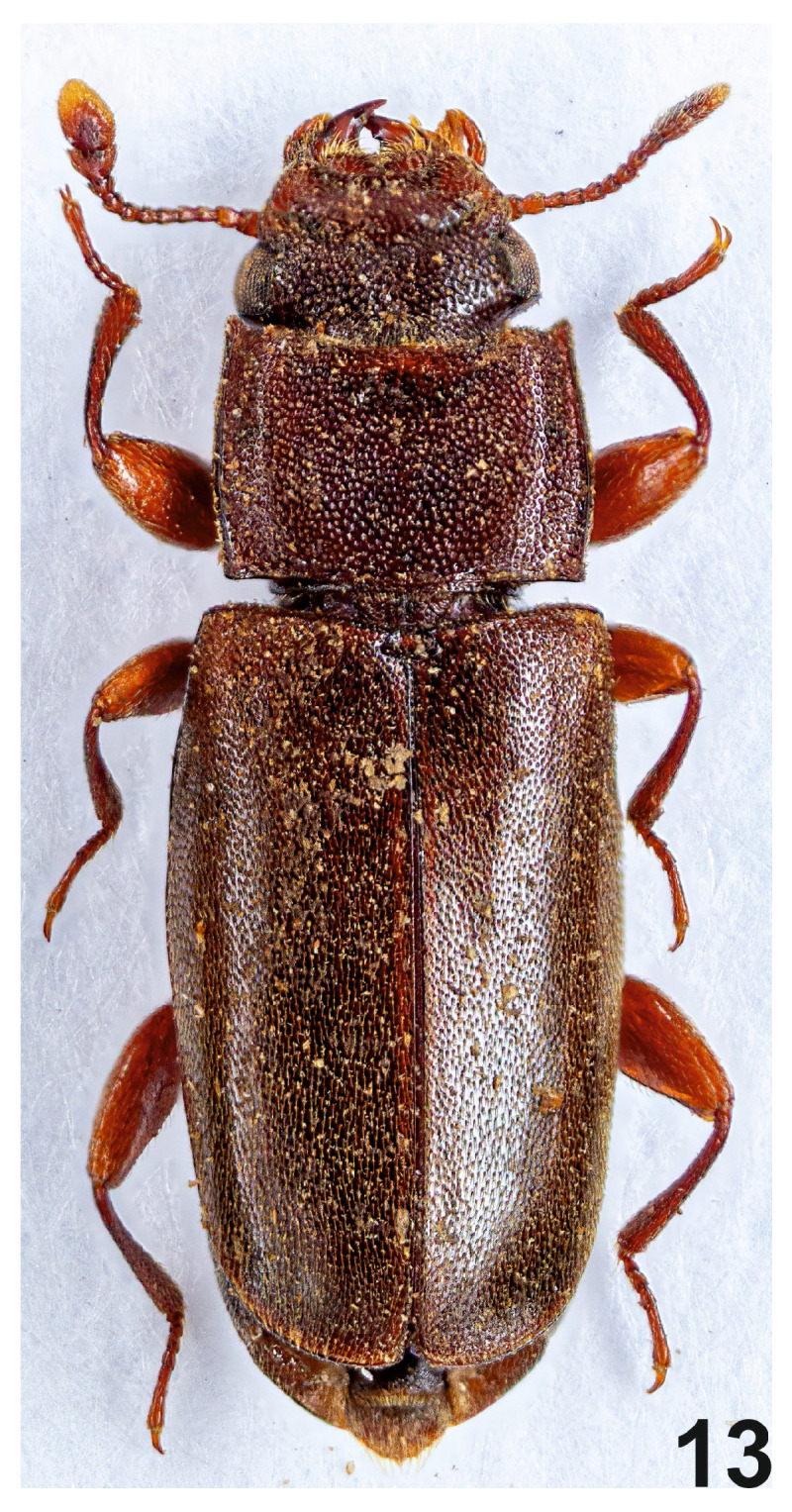
*Cephalotoma patcharinae* Borowski n. sp., dorsal view.

Because of the two-segmented club of antennae (Figure 1(4)), the new species is very easily differentiated from other representatives of the genus that occur in the Oriental region and has a three-segmented club of antennae (Figure 2(7–10)). *C. patcharinae* Borowski n. sp. can be easily differentiated from African species, which also have a two-segmented club of antennae (Figure 2(11,12)) by comparing the length of the last two segments of the antennae club; in the new species, the last segment is twice as long as the penultimate one, while in the African species, both segments are of equal length. The almost matt, densely setose surface of the elytra of the new species is also characteristic. Only *C. singularis* has elytra that are weakly shiny, densely setose, and, in this respect, may somewhat resemble the new species. The remaining species, including the African ones, have lustrous elytra and are usually indistinctly setose. The shape of the pronotum differs between *C. singularis* and the new species. In the new species, the greatest width of the pronotum is just behind its mid-length (Figure 1(3) and Figure 3), while in *C. singularis*, the greatest width is located near the anterior angles of the pronotum.

### 3.3. Name Derivation

The new species was named after our colleague, Dr. Patcharin Krutmuang (Department of Entomology and Plant Pathology, Chiang Mai University, Thailand), as a token of gratitude for her care during the Polish scientific team’s stay in Thailand.

### 3.4. Biology

The specimens of the new species were taken from the galleries of *Xylothrips flavipes* (Illiger, 1801) (Bostrichidae, Xyloperthini) located in a branch about 2 cm thick near an unspecified deciduous tree; the branches of a felled tree were on a roadside, near a coffee plantation.

## 4. Discussion and Conclusions

Liu and Schönitzer [9] created a new tribe called Cephalotomini, into which they classified two genera: *Cephalotoma* and *Lyctoderma*. A morphological analysis covered representatives of two genera (*Cephalotoma*, *Trogoxylon*) out of six then known. In Table 1, we present a description of the Cephalotomini tribe derived from the paper by Liu and Schönitzer [9], along with relevant comments for the described features.

Considering the above, it can be clearly seen that the species classified as Cephalotomini only slightly differ from the other representatives of Trogoxylini. Perhaps some concept of Trogoxylini division is right, e.g., division into subtribes, but an analysis should require the knowledge of all the Trogoxylini genera, and not only two of them, as was presented in the paper by Liu and Schönitzer [9]. Due to the morphological traits presented above, which are almost completely characteristic of the representatives of the other Trogoxylini genera and even Lyctinae, we synonymise the tribe Cephalotomini n. syn. with Trogoxylini.

## 5. Key for the Identification of Oriental *Cephalotoma* Species (The Key for Identifying the Remaining World Species of the Genus *Cephalotoma* Can Be Found in the Book of Borowski and Węgrzynowicz [2])

1. Antennal club two-segmented (Figure 1(4))………………………………………............... ………………………………………………*C. patcharinae* Borowski n. sp. (N. Thailand)
  - Antennal club three-segmented (Figure 2(7–10))........................................................2.
2. Front and pronotum glabrous. Median depression on pronotum conspicuous. Pronotum widest at mid-length...........................*C. perdepressa* Lesne (Vietnam, Sri Lanka)
  - Front and pronotum setose. Pronotal disk flat. Maximum pronotal width at anterior margin……….................................................................................................3.
3. Pronotal sides without fine oblique wrinkles. Punctures on pronotum equally dense all over its surface, median line undifferentiated. Elytral puncturation dense, surface matte or faintly shining, distinctly setose.......................................................................... …………………*C. singularis* Lesne (Indochinese Peninsula, Indonesia, New Guinea)
  - Pronotal sides finely obliquely wrinkled. Punctures on pronotum denser on sides and sparser on disk, with indistinct median line. Eytral surface more or less lustrous, almost glabrous or with conspicuous setae......................................4.
4. Elytral surface strongly lustrous, almost glabrous, at most near apices some very short setulae may occur. On clypeus and front setae short, not reaching bases of those before them.........................................*C. coomani* (Lesne) (Indochinese Peninsula).
  - Elytral surface slightly shining, all covered with conspicuous recumbent setae. On clypeus and front setae long, reaching bases of those before them.................................................................................*C. ambiguum* (Lesne) (India).

## Figures and Tables

**Table 1:** Characters of tribe Cephalotomini defined by Liu and Schönitzer [9] with comments and remarks.

Character	Comments and Remarks
1. Body very flattened, less elongated than other lyctines	Not true: the genera *Tristaria* Reitter, 1878 and *Phyllyctus* Lesne, 1911 have the same shape of body
2. Eyes large and very strongly projecting, occupying 40% of total head width	The genus *Phyllyctus* or *Trogoxylyctus* Węgrzynowicz and Borowski, 2015 [11] have identical eyes
3. Clypeus lobed or dentate and its anterior angles, emarginated in front	All species of Trogoxylinii have more or less emarginated clypeus, often lobed or dentate
4. Clypeo-frontal suture obsolescent, and clypeus and frons lie in almost the same plane, unlike other lyctines	Not true: clypeo-frontal suture is usually distinctly visible in all genera of Troxylini, e.g., in *Phyllyctus,* is identical to *Cephalotoma*
5. Labrum bilobate, fringed with fine and long hairs	All species of Lyctinae have bilobate, fringed with fine and long hairs
6. Gular suture indistinct with a wide and impunctate gular area	All species of Trogoxylini have gular suture indistinct with a wide and impunctate gular area
7. Antenna 11 segmented, club 3-segmented (except for African species with 2-segmented club)	Incorrectly selected character
8. Pronotum more transverse than in other lyctines, with distinct lateral margins, anterior and posterior angles distinct	Lateral margins, anterior and posterior angles are distinct in *Cephalotoma*, *Phyllyctus*, *Trogoxylyctus* and partly in *Trogoxylon*
9. Elytra flat, more or less parallel, without declivity and epipleura, without striae	Typical characters for all species of Trogoxylini
10. Legs moderately long, stout; first tarsomere very small, fifth tarsomere almost as long as all preceding segments combined	Typical characters for all species of Trogoxylini
11. Coxae widely separated, pro- and meso-coxae rounded, and weakly projecting	Typical characters for all species of Trogoxylini
12. Intercoxal process of first visible abdominal sternite broadly truncates anteriorly	Typical character for almost all species of Trogoxylini

## Data Availability

Material used in this work are available in the collections of the Department of Forest Protection, SGGW, Warsaw, Poland.
